# Phenotypic and Genotypic Characterization of Animal-Source* Salmonella* Heidelberg Isolates

**DOI:** 10.1155/2016/6380890

**Published:** 2016-01-03

**Authors:** Kristin A. Clothier, Barbara A. Byrne

**Affiliations:** ^1^California Animal Health & Food Safety Lab System, School of Veterinary Medicine, University of California, Davis, Davis, CA 95616, USA; ^2^Departments of Pathology, Microbiology, and Immunology, School of Veterinary Medicine, University of California, Davis, Davis, CA 95616, USA; ^3^Veterinary Medical Teaching Hospital, School of Veterinary Medicine, University of California, Davis, Davis, CA 95616, USA

## Abstract

*Salmonella enterica* serotype Heidelberg (*S.* Heidelberg) is frequently implicated in human foodborne* Salmonella* infections and often produces more severe clinical disease than other serotypes. Livestock and poultry products represent a potential risk for transmission to humans. The purpose of this study was to evaluate 49* S.* Heidelberg veterinary isolates for exponential growth rate (EGR), PFGE pattern, and antimicrobial resistance to evaluate these parameters as mechanisms by which* S.* Heidelberg emerged as a virulent foodborne pathogen. Isolates were categorized by species of origin; clinical or environmental sources; and time frame of recovery. Growth rates were determined in nutrient media using serial dilutions and colony counts; PFGE was performed according to the CDC PulseNet protocol. Minimum inhibitory concentration and susceptibility determinations were performed against antimicrobials important in human medicine. Eighteen unique PFGE patterns were detected in the isolates tested. Antimicrobial resistance was significantly greater (*P* < 0.05) for ten of 15 drugs in clinical over environmental isolates; for four drugs between the time frames; and for ten drugs between species of origin. The large genetic diversity present in isolates of this serotype may convey competitive advantages to this organism, while the presence of antimicrobial resistance represents a potential zoonotic risk via animal-source food products.

## 1. Introduction

*Salmonella* is a major cause of foodborne outbreaks in the USA [1–4]. In 2012 alone,* Salmonella* was implicated in 25% of the 423 outbreaks associated with an infectious agent [2].* S. enterica* serotype Heidelberg (*S.* Heidelberg) consistently ranks in the top ten serotypes detected from laboratory-confirmed* Salmonella* infections [2, 5–7]. Additionally,* S*. Heidelberg has been recovered from many livestock and poultry species posing risks for zoonotic transmission via food products [8–10].

Infections with* S.* Heidelberg are more likely to result in severe disease than other serotypes, emphasizing its pathogenic potential [7, 11, 12]. Recently, poultry products were implicated in* S.* Heidelberg outbreak that resulted in 634 illnesses involving people from 29 states [13]. While most foodborne* Salmonella* infections are self-limiting, 38% of patients in this outbreak required hospitalization [13]. Several published reports have documented a high prevalence of antimicrobial resistance in isolates of this serotype to a variety of drugs, further highlighting the potential health risk that* S*. Heidelberg represents [11, 14, 15].

Reasons for the emergence of* S.* Heidelberg as a virulent pathogen are unknown but may include phenotypic or genotypic changes over time that have enhanced fitness over other bacterial agents. Microbial growth rates are utilized in evaluating the effects of treatment on food products to determine the likelihood of risk reduction for specific interventions [16]. Higher growth rates decrease time needed to reach an infectious dose which may convey enhanced fitness to bacterial populations exhibiting them. Serotyping is essential part of* Salmonella* epidemiologic investigations; however, molecular subtyping methods can provide more insight into isolate relatedness within serotypes [17]. Pulse field gel electrophoresis (PFGE) is a highly robust method for assessing DNA similarity between isolates with high reproducibility between laboratories and is the preferred method for surveillance and outbreak investigations [18]. A standardized PFGE protocol published by CDC is widely used for foodborne* Salmonella* investigations [5, 19]. The purpose of the present study was to evaluate a panel of* S.* Heidelberg isolates recovered from veterinary clinical and environmental sources for exponential growth rate (EGR), PFGE pattern, and antimicrobial resistance over time and by source and species of origin to evaluate alterations in these parameters as mechanisms by which* S.* Heidelberg may have emerged as a highly virulent foodborne pathogen.

## 2. Materials and Methods

### 2.1. Bacterial Isolates

Forty-nine* S.* Heidelberg isolates were randomly selected for this study from the historical collection of bacteria recovered from samples submitted to the California Animal Health & Food Safety Lab System (CAHFS) from 1991 to 2013. Isolates had been stored at −70°C in preservation solution (Microbank, Pro-Lab Diagnostics, Austin, TX) or lyophilized for long-term storage as previously described [20]. Isolates were categorized according to animal species of origin (bovine [*n* = 9], chicken [*n* = 34], equine [*n* = 2], and turkey [*n* = 4]); associated history (recovered from clinical cases [*n* = 21] or environmental sources [*n* = 28]); and time frame of recovery (prior to 2006 [*n* = 11], 2006–2011 [*n* = 12], and 2012-2013 [*n* = 26]). Isolates from clinical cases were recovered from animals with diarrhea (chicken, bovine, and equine), nonenteric infections including peritonitis, pericarditis, hepatitis, pneumonia, and sepsis (chicken, turkey, and bovine), and gastrointestinal stasis (equine). Environmental sources consisted of drag swabs, fecal samples from animal lounging sites, rinse water, chicken fluff samples, and bedding.

### 2.2. EGR Determination

Bacterial isolates were recovered from long-term storage onto nutrient agar (5% sheep blood agar, SBA) and incubated at 35–37°C for 18–24 hours. Serotype identity was confirmed by biochemical and serological testing. Culture plates were assessed for purity and subcultured into liquid brain-heart infusion broth (BHI) incubated at 35–37°C for 18–24 hours prior to testing. Growth curve experiments were performed using published protocols [16]. Briefly, isolates were grown in flasks containing 150 mL of BHI incubated in ambient air at 35–37°C on a shaking incubator at 120 rpm. Aliquots were collected at 0, 2, 4, 6, and 8 hours of incubation and colony forming units per milliliter (cfu/mL) counts were determined using serial dilutions and culture plating on SBA. Exponential growth rates (EGR)* in vitro* were assessed utilizing published standard methods [21, 22] and calculated using *y* = *be* ^*Ax*^, where *y* is the final concentration of bacteria in the culture, *b* is the initial concentration in the culture, *A* is the exponential growth rate, and *x* is the time of incubation. Summary statistics (mean, standard deviation [SD], and coefficient of variation [CV]) were calculated by species of origin, associated source, and time frame of recovery.

### 2.3. PFGE Testing

PFGE was performed according to the PulseNet protocol developed by the CDC utilizing digestion with* Xba*I (Promega) and analyzed using BioNumerics software (Applied Maths, Inc., Austin, TX) at the Laboratory for Molecular Typing, Cornell University. Isolates were considered identical if they differed by 0-1 bands, potentially related if they differed by 2-3 bands, and likely unrelated if they differed by >3 bands [17].

### 2.4. Antimicrobial Susceptibility Testing

Minimum inhibitory concentration (MIC) values were determined using microbroth dilution methods using TREK Sensititre (Thermo Fisher Scientific, Pittsburgh, PA) according to published criteria [23]. Testing was performed using the National Antimicrobial Resistance Monitoring System (NARMS) antimicrobial susceptibility panel. Susceptibilities were determined for each isolate against amoxicillin/clavulanic acid (AMC, 1/0.5–32/16 *μ*g/mL), ampicillin (AMP, 1–32 *μ*g/mL), azithromycin (AZI, 0.12–16 *μ*g/mL), cefoxitin (FOX, 0.5–32 *μ*g/mL), ceftiofur (TIO, 0.12–8 *μ*g/mL), ceftriaxone (AXO, 0.25–64 *μ*g/mL), chloramphenicol (CHL, 2–32 *μ*g/mL), ciprofloxacin (CIP, 0.015–4 *μ*g/mL), gentamicin (GEN, 0.25–16 *μ*g/mL), kanamycin (KAN, 8–64 *μ*g/mL), nalidixic acid (NAL, 0.5–32 *μ*g/mL), streptomycin (STR, 32–64 *μ*g/mL), sulfisoxazole (SUL, 16–256 *μ*g/mL), tetracycline (TET, 4–32 *μ*g/mL), and trimethoprim-sulfamethoxazole (SXT, 0.12/2.4–4/76 *μ*g/mL).* Escherichia coli* American Type Culture Collection (ATCC) 25922,* E. coli* ATCC 35218,* Enterococcus faecalis* ATCC 29212,* Pseudomonas aeruginosa* ATCC 27853, and* Staphylococcus aureus* ATCC 29213 were used as quality control organisms. Susceptibility determinations were established using CLSI criteria where available [24]. Antimicrobials for which there are no CLSI interpretive criteria (AZI and STR) were evaluated using USDA NARMS guidelines [19]. Percent of resistant isolates was calculated as the number of isolates classified as “resistant” divided by the total number of isolates tested expressed as a percentage.

### 2.5. Statistical Analysis

Exponential growth was assessed for significant differences (*P* < 0.05) in mean rate between source, time frame of recovery, and species of origin using a one-way ANOVA. Percent of isolates resistant to an individual antimicrobial was assessed for statistically significant differences (*P* < 0.05) based on the null hypothesis that resistance was consistent between time frame of recovery, sample source, and species of origin using Fisher's exact test. Mode MIC value and range were determined for each antimicrobial by source, time frame, and species of origin; the nonparametric Kruskal-Wallis test was used to compare MICs for significant (*P* < 0.05) differences between groups for each of these categories. Statistical evaluations were performed using SAS Version 9.4 (SAS Institute, Inc., Cary, NC, USA).

## 3. Results

### 3.1. EGR Determination

Table 1 presents the summary statistics for EGR of these isolates by source, time frame of recovery, and species of origin, as well as the number of unique PFGE patterns for these criteria. The starting bacterial concentrations ranged from 7 × 10^1^ to 4 × 10^4^ cfu/mL and reached 2 × 10^8^ to 8 × 10^9^ cfu/mL after eight hours of incubation. EGR ranged from 1.151 to 2.105/hr. No significant differences in growth parameters were identified between time periods (*P* = 0.65), between species of origin (*P* = 0.68), or between clinical and environmental sources (*P* = 0.19).

### 3.2. PFGE Evaluation

**Figure 1:**
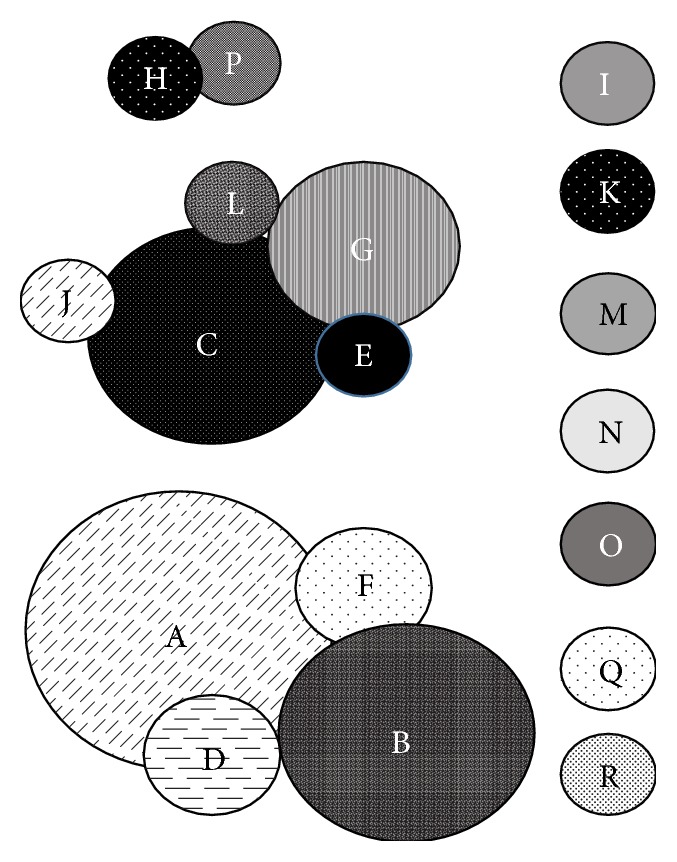
Relative relatedness (intersecting circles represent patterns that differ by ≤3 bands) and relative prevalence (size of circle denotes the frequency of identical patterns) of PFGE patterns determined for 49* Salmonella* Heidelberg isolates recovered from CAHFS from 1991–2013. Letters designate individual PFGE patterns (A = 13, B = 9, C = 7, D = 2, E = 1, F = 2, G = 4, and H–R = 1 isolate each).

Eighteen distinct PFGE patterns were recognized in this group of isolates. Three patterns were identified more frequently than the others, primarily in environmental samples (A: *n* = 13 total, 8 from environmental samples; B: *n* = 9 total, 8 from environmental samples; C: *n* = 7 total, 6 from environmental samples). While these same three patterns were more commonly identified in samples from 2012-2013, eight other patterns were detected in this group as well.  Figure 1 shows the relative relatedness (intersecting circles represent patterns that differ by ≤3 bands) of isolates determined by PFGE pattern analysis along with the relative prevalence of each pattern (size of circle). Isolates clustered into three potentially related groups (2-3 bands different from one another) and into seven unique patterns (>3 bands different from one another) with a single isolate in each.

### 3.3. Antimicrobial Susceptibility Evaluation

**Figure 2:**
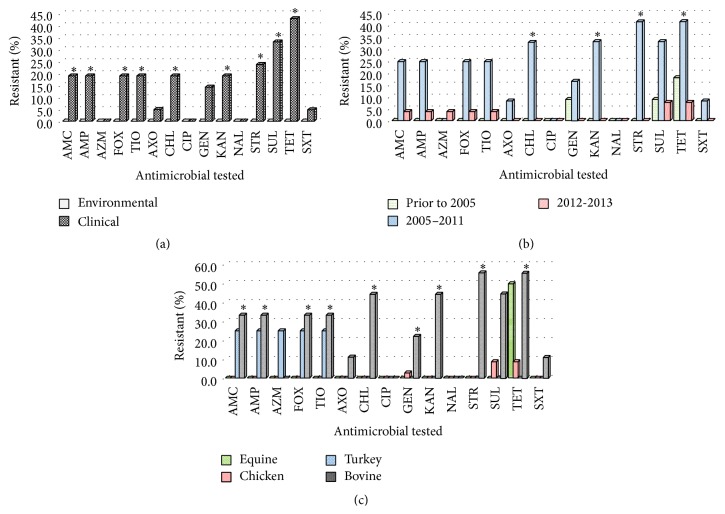
Percentage of resistance to the listed antimicrobials in* Salmonella* Heidelberg isolates from samples recovered (a) from environmental and clinical samples, (b) prior to 2006, 2006–2011, and 2012-2013, and (c) from equine, chicken, turkey, and bovine specimens. AMC = clavulanic acid/ampicillin, AMP = ampicillin, AZI = azithromycin, FOX = cefoxitin, TIO = ceftiofur, AXO = ceftriaxone, CHL = chloramphenicol, CIP = ciprofloxacin, GEN = gentamicin, KAN = kanamycin, NAL = nalidixic acid, STR = streptomycin, SUL = sulfisoxazole, TET = tetracycline, and SXT = trimethoprim/sulfamethoxazole; **∗** = significantly different (*P* < 0.05) percent of resistant isolates between (a) source, (b) time frame of recovery, and (c) species of origin.

The percentages of resistant isolates by source (panel (a)), time frame of recovery (panel (b)), and species of origin (panel (c)) are shown in Figure 2. There was significantly (*P* < 0.05) greater resistance in clinical isolates to AMC (19.0%), AMP (19.0%), FOX (19.0%), TIO (19.0%), AXO (19.0%), CHL (19.0%), KAN (19.0%), STR (23.8%), SUL (33.3%), and TET (42.9%) over environmental sources, which showed no resistance to any of the tested drugs. Antimicrobial resistance was significantly different (*P* < 0.05) between time frames of recovery for four compounds, with the greatest percent of resistant isolates for CHL (33.0%), KAN (33.0%), STR (41.7%), and TET (41.7%) in isolates recovered between 2005 and 2011.

Overall, resistance in these isolates was low for equine samples (one isolate to TET) and chicken samples (one isolate to GEN [2.9%], three isolates to SUL [8.8%], and three isolates to TET [8.8%]). One of the four turkey samples was resistant to multiple drugs (AMC, AMP, AZI, FOX, and TIO). Statistically significant differences (*P* < 0.05) were identified between species of origin for AMC, AMP, FOX, TIO, AXO, CHL, KAN, STR, and TET, with bovine isolates exhibiting the highest percent of resistant isolates to these antimicrobials (AMC [33.3%], AMP [33.3%], FOX [33.3%], TIO [33.3%], AXO [33.3%], CHL [44.4%], KAN [44.4%], STR [55.6%], and TET [55.5%]).

### 3.4. MIC Results

Mode MIC values as well as the range of MICs for the isolates by source, time frame, and species of origin are presented in Table 2. Clinical isolates demonstrated significantly (*P* < 0.05) greater MICs for AMC, AMP, TIO, AXO, GEN, KAN, STR, SUL, and TET. Significant differences in MICs were identified between time frames for AXO, STR, and TET with a greatest percentage of isolates at the high end of the MIC range from 2005 to 2011 and between species for AMC, AMP, FOX, TIO, AXO, CIP, KAN, STR, and TET, in which equine and turkey isolates had the highest MICs for CIP while bovine isolates had the highest MIC for the remaining antimicrobials.

## 4. Discussion

Isolates studied in the present work demonstrated large genetic diversity, with seven PFGE patterns that had >3 band differences from all other patterns. Isolates are considered to be potentially closely related or share a common ancestor when pattern differences are consistent with a single genetic event, demonstrated by two to three band differences [17]. Pattern A, the most commonly identified pattern (*n* = 13), contained isolates from bovine, chicken, and turkey samples from clinical and environmental sources and from all three time frames. Patterns B (*n* = 9) and C (*n* = 7) were all from poultry sources but were also collected from all time frames and from clinical and environmental sources. Genetic variation such as that seen in these isolates can provide selective advantage, particularly during times of environmental stress [25]. Altered patterns of gene expression convey the ability to withstand stressful conditions such as extremes of heat and host immune response [25] which could provide competitive advantage to this serotype.

Bacterial exponential growth rates and the resultant doubling times are considered a species-specific characteristic under equivalent conditions; however, the acquired genes or mutations over time could provide additional advantage and alter exponential growth rate [22]. Reported doubling times in* S.* Typhimurium strains have been estimated at 27 to 30 minutes under nutrient-rich culture conditions [26, 27]. Doubling time for the isolates in this study was very consistent, ranging from 19.7 to 24.5 minutes for 47 of the 49 isolates studied, and differences over time or between sources were not identified. Although other serotypes were not investigated in the present work, it is possible that growth rates vary across* Salmonella* serotypes and faster growth rates may provide* S.* Heidelberg with a competitive advantage at similar infectious doses.

Four multidrug resistant (>5 drugs) isolates were identified in this collection (3 bovine animals recovered, 2006–2011, and 1 turkey recovered, 2012-2013; 8.2% of total tested). Isolates were resistant to AMC (*n* = 4), AMP (*n* = 4), FOX (*n* = 4), TIO (*n* = 4), CHL (*n* = 3, all bovine), KAN (*n* = 3, all bovine), STR (*n* = 3, all bovine), and TET (*n* = 3, all bovine). None of these isolates was recovered from animals with any herd or geographic relatedness. The presence of multidrug resistant isolates is of concern particularly to antimicrobials critical in the treatment of human infections. Antimicrobial resistance genes are most commonly carried on plasmids [7], and future testing for the presence of specific resistance genes as well as known plasmids may give more insight into the ecology of resistance found in these strains. Interestingly, all of the isolates recovered from animal environmental sources were susceptible to all antimicrobials tested, even those from sites containing feces from large numbers of animals (drag swabs, lounging areas). While antimicrobial use would be more likely in animals with clinical disease, particularly those with systemic symptoms, data on drug use for treatment or metaphylaxis in these animals was not provided. Consequently, establishing the risks of prior treatment on the presence of antimicrobial resistance was beyond the scope of this study.

Differences in MIC values even within the “susceptible range” can give early indications of trends toward resistance development and potential treatment failure [28]. Significant differences in MICs in this study closely followed those for percent of resistant isolates for many drugs; however, MICs for certain antimicrobials even within the susceptible range did demonstrate patterns for concern, specifically differences in ceftiofur (*P* = 0.043) and ciprofloxacin (*P* = 0.0008) between species and gentamicin (*P* = 0.025) between sources. Alterations in MIC of a single dilution are often considered biologically insignificant; however, epidemiologic studies on clinical treatment outcomes have determined that this may not be accurate. A study by Sakoulas et al. [29] identified a statistically significant difference (*P* < 0.02) in treatment success against methicillin-resistant* S. aureus* between vancomycin isolates with a MIC of ≤0.5 *μ*g/mL and those with a MIC of 1-2 *μ*g/mL even though the breakpoint for susceptible is ≤2 *μ*g/mL. Assessments for MIC differences between groups can provide information that may not be evident when only looking at patterns of resistance.

Several limitations are evident in the present study, including the use of a convenience sample of isolates recovered in a single diagnostic laboratory system which would not be expected to represent the status of all* S.* Heidelberg isolates present in food-producing animals. Only two equine-source isolates were evaluated in this survey, limiting the value of conclusions about* S.* Heidelberg from this species. Additionally, the antimicrobials studied are important in human clinical use and not utilized in food-producing animals; consequently, the patterns found in these isolates may not fully demonstrate all resistance present in these bacteria.

Data from this work demonstrates that while growth rates were consistent within* S.* Heidelberg isolates from animals, genetic diversity was high which facilitate bacterial response to stress and agent survival. Although antimicrobial resistance was not widespread, the percent of resistance to a variety of drugs is of concern due to the risks of contamination of animal-source food products. Assessments for mechanisms of resistance were beyond the scope of this study, but future work investigating the presence of known resistance genes or acquired efflux mechanisms, particularly in the multidrug resistant isolates in this group, may reveal reasons for resistance development. Investigations on field isolates like those evaluated here can provide valuable insight into the potential risks from zoonotic pathogens which may be spread via animal food products.

## Figures and Tables

**Table 1:** Summary statistics on exponential growth rate (EGR) measurements and number of unique PFGE patterns for *Salmonella* Heidelberg isolates by isolate source, time frame of recovery, and species of origin.

	Exponential growth rate (EGR)			Number of unique PFGE patterns
	Mean	SD	CV	
Isolate source				
Clinical (*n* = 21)	1.84	0.18	0.10	14
Environmental (*n* = 28)	1.90	0.13	0.07	8
Time frame of isolation				
Prior to 2006 (*n* = 11)	1.90	0.08	0.04	6
2006–2011 (*n* = 12)	1.88	0.09	0.05	9
2012-2013 (*n* = 26)	1.86	0.19	0.10	11
Species of origin				
Chicken (*n* = 34)	1.87	0.18	0.09	12
Bovine (*n* = 9)	1.83	0.06	0.03	6
Turkey (*n* = 4)	1.92	0.08	0.04	3
Equine (*n* = 2)	1.94	0.11	0.06	2

PFGE = pulse field gel electrophoresis; SD = standard deviation; CV = coefficient of variation.

**Table 2:** Summary statistics of minimum inhibitory concentrations (MIC) values for *S. *Heidelberg isolates by source, time frame of recovery, and species of origin.

ABTC	MIC by source		MIC by time frame			MIC by species			
	(*µ*g/mL)		(*µ*g/mL)			(*µ*g/mL)			
	CLIN	ENV	<2006	2006–2011	2012-2013	BO	CH	TU	EQ^†^
	*n* = 21	*n* = 28	*n* = 11	*n* = 12	*n* = 26	*n* = 9	*n* = 34	*n* = 4	*n* = 2
AMC									
Mode	1/0.5^a^	1/0.5	1/0.5	1/0.5	1/0.5	1/0.5^c^	1/0.5	1/0.5	1/0.5
Range	1/0.5–32/16	1/0.5	1/0.5	1/0.5–32/16	1/0.5–32/16	1/0.5–32/16	1/0.5–2/1	1/0.5–32/16	1/0.5
AMP									
Mode	1^a^	1	1	1	1	1^c^	1	1	1
Range	1–32	1-2	1-2	1–32	1–32	1–32	1-2	1–32	1
AZI									
Mode	4	4	4	4	4	4	4	4	8
Range	2–16	2–8	4–8	2–8	2–16	2–8	2–8	4–16	4–8
FOX									
Mode	2	2	2	2	1	32^c^	2	2	4
Range	0.5–32	1-2	1–4	0.5–32	1–32	0.25–32	1-2	2–32	2–4
TIO									
Mode	1^a^	0.5	1	0.5	0.5	8^c^	0.5	1	1
Range	0.25–8	0.5–1	0.5–1	0.5–8	0.5–8	0.25–8	0.5–1	1–8	0.5–1
AXO									
Mode	0.25^a^	0.25	0.25^b^	0.25	0.25	0.25^c^	0.25	0.25	0.25
Range	0.25–64	0.25	0.25	0.25–64	0.25–32	0.25–64	0.25	0.25–32	0.25
CHL									
Mode	4	4	8	4	5	4	4	8	8
Range	4–32	4–8	4–8	4–32	4–16	4–32	4–8	4–16	4–8
CIP									
Mode	0.015	0.015	0.015	0.015	0.015	0.015^c^	0.015	0.015	0.03
Range	0.015–0.03	0.015	0.015–0.03	0.015	0.015–0.03	0.015	0.015	0.015–0.03	0.015–0.03
GEN									
Mode	0.5^a^	0.5	0.5	0.5	0.5	0.5	0.5	0.5	1
Range	0.5–16	0.25–8	0.5–16	0.5–16	0.25–8	0.5–16	0.25–16	0.5	0.5–1
KAN									
Mode	8^a^	8	8	8	8	8^c^	8	8	8
Range	8–64	8	8–32	8–64	8–32	8–64	8–32	8	8
NAL									
Mode	4	4	4	2	4	4	4	4	4
Range	2–4	2–4	2–4	2–4	2–4	2–4	2–4	2–4	2–4
STR									
Mode	32^a^	32	32^b^	32	32	32^c^	32	32	32
Range	32–64	32	32	32–64	32	32–64	32	32	32
SUL									
Mode	1^a^	1	1	1	1	1	1	1	128
Range	1–256	1–128	1–256	1–256	1–256	1–256	1–256	1	1–128
TET									
Mode	4^a^	4	4^b^	4	4	32^c^	4	4	32
Range	4–32	4	4–32	4–32	4–32	4–32	4–32	4	4–32
SXT									
Mode	0.12	0.12	0.12	0.12	0.12	0.12	0.12	0.12	0.12
Range	0.12–4	0.12–2	0.12	0.12–4	0.12–2	0.12–4	0.12–2	0.12	0.12

ABTC = antimicrobial tested; CLIN = clinical, ENV = environmental; CH = chicken; BO = bovine; EQ = equine; TU = turkey; AMC = amoxicillin-clavulanic acid, AMP = ampicillin, AZI = azithromycin, FOX = cefoxitin, TIO = ceftiofur, AXO = ceftriaxone, CHL = chloramphenicol, CIP = ciprofloxacin, GEN = gentamicin, KAN = kanamycin, NAL = nalidixic acid, STR = streptomycin, SUL = sulfisoxazole, TET = tetracycline, and SXT = trimethoprim/sulfamethoxazole; ^†^Mode MIC was listed to the greater of the two values; ^a^MIC values are significantly (*P* < 0.05) different between sources; ^b^MIC values are significantly (*P* < 0.05) different between time frames; ^c^MIC values are significantly (*P* < 0.05) different between species.
